# ELISA assay employing epitope-specific monoclonal antibodies to quantify circulating HER2 with potential application in monitoring cancer patients undergoing therapy with trastuzumab

**DOI:** 10.1038/s41598-020-59630-y

**Published:** 2020-02-20

**Authors:** Valentina Agnolon, Anna Contato, Anna Meneghello, Elda Tagliabue, Giuseppe Toffoli, Massimo Gion, Federico Polo, Aline S. C. Fabricio

**Affiliations:** 1 Regional Center for Biomarkers, Department of Clinical Pathology and Transfusion Medicine, Azienda ULSS 3 Serenissima, Regional Hospital, Campo SS Giovanni e Paolo 6777, 30122 Venice (VE), Italy; 2 Experimental and Clinical Pharmacology, Centro di Riferimento Oncologico (CRO) IRCCS, Via F. Gallini 2, 33081 Aviano (PN), Italy; 3 Molecular Targeting Unit, Fondazione IRCCS Istituto Nazionale dei Tumori, Via Venezian 1, 20133 Milan (MI), Italy; 4 Present Address: Division of Immunology and Allergy, Centre Hospitalier Universitaire Vaudois (CHUV), Rue du Bugnon 46, 1011 Lausanne, Switzerland; 5 Present Address: Department of Molecular Sciences and Nanosystems, Ca’ Foscari University of Venice, Via Torino 155, 30172 Venezia, Italy

**Keywords:** ELISA, Breast cancer, Clinical pharmacology, Biomarkers

## Abstract

Circulating HER2 extracellular domain (HER2 ECD) levels were proposed as a surrogate for HER2 tissue expression to monitor breast cancer patients for early relapse or responses to standard or HER2-targeted therapies, such as the monoclonal antibody (mAb) trastuzumab. Currently, available commercial ELISA assays for HER2 ECD rely on antibodies recognizing undisclosed or unknown epitopes. In this work, two ELISA assays employing MGR2 and MGR3 epitope-specific mAbs for HER2 ECD were developed and validated, showing good assay precision and linearity of the dose-response signal within the dynamic range of 0.19–12.50 ng mL^−1^ and detection limits of 0.76 and 0.75 ng mL^−1^ for the MGR2 and MGR3 assays, respectively. The developed assay showed a good agreement with two widely used commercial kits for HER2 ECD quantification in serum samples from breast cancer patients. A complete characterization of mAb-HER2 ECD interaction was performed by means of surface plasmon resonance using trastuzumab as control for both epitope mapping and kinetics analysis. The epitopes recognized by the two mAbs showed no overlap with trastuzumab, which was confirmed by trastuzumab interference analysis in serum samples. The method showed to be a practical approach to determine HER2 ECD with a high degree of sensitivity, reliability and recovery in samples containing mAbs-based therapies.

## Introduction

HER2 is a trans-membrane tyrosine kinase receptor, member of the epidermal growth factor receptor (EGFR) family. Abnormal expression of HER2 was observed in a number of primary tumors, including breast cancer (BC) and gastric cancer, where HER2 gene is overexpressed in approximately 30% and 24% of patients, respectively^1,2^. Determination of HER2 overexpression in BC tissue has a role in clinical decisions, as it is associated with an unfavourable prognosis, especially in patients with lymph node metastases^3^, with greater resistance to hormonal therapy and some chemotherapeutical agents^4,5^ and with increased sensitivity to other chemotherapy regimens^6,7^. Moreover, HER2 protein tissue expression has been employed as an important predictive factor to take decision about anti-HER2 therapeutic approaches such as the use of monoclonal antibodies (mAbs) trastuzumab (Herceptin™, Genentech) and pertuzumab (Perjeta™, Genentech)^8^. The combination of trastuzumab with adjuvant chemotherapy is the current standard of treatment for HER2-positive patient population as it markedly improved outcomes among patients with HER2-positive early BC, reducing the risk of disease relapse and death^9–12^.

HER2 is a 185 kDa protein composed of three domains: a cytoplasmic domain with catalytic activity, a single transmembrane domain, and an extracellular (ECD) ligand-binding domain containing 647 amino acids. ECD is composed of four subdomains (I, II, III and IV) arranged as a tandem repeat of a two-subdomain unit, which can be cleaved from the surface of breast cancer cells by specific matrix metalloproteases^13–15^.

HER2 status of patients with BC and their eligibility for trastuzumab therapy have been determined using tissue-based tests such as immunohistochemistry (IHC) and fluorescent *in situ* hybridization (FISH)^16^. However, these histological techniques requiring invasive biopsies are indicated to assess HER2 status in the primary tumor but cannot be used in the follow-up to monitor the disease and the response to treatment^17,18^. In this context, the detection of circulating HER2 ECD levels was proposed as a surrogate marker in metastatic BC (MBC) for a less invasive analysis of HER2 status in tissue^18^. In fact, serum HER2 ECD is currently suggested in the follow-up and monitoring of patients with MBC, whose initial serum HER2 value is above 15 ng mL^−1^ ^19^, as high serum HER2 levels in MBC patients correlate with poor prognosis and decreased response to both conventional (hormone-therapy and taxane) and anti-HER2 targeted (i.e. pertuzumab, trastuzumab) anticancer therapies. However, available methods are not sufficiently sensitive for HER2 ECD determination in primary BC and during the treatment follow-up to identify acquired resistance to HER2-targeted antibody therapy. Moreover, it is impossible to compare results among studies evaluating the predictive role of HER2 ECD due to the lack of standardisation among the different immunoassays used (different cut-offs, different monoclonal antibodies with non-defined specificities).

In the present study we describe the development of sensitive enzyme-linked immunosorbent assays (ELISA), based on two epitope-specific monoclonal antibodies, namely MGR2 and MGR3, which were capable of correctly quantifying HER2 ECD also in presence of trastuzumab. The latter antibodies were developed in house and selected with the aim of further implementing applications in the clinical practice. Considering that trastuzumab can bind soluble HER2 ECD, its quantification through antibodies that (i) are fully characterized and (ii) recognise non-overlapping epitopes is crucial. Two sandwich ELISAs based on MGR2 and MGR3 epitope-specific mAbs for HER2 ECD were developed and validated. These mAbs were obtained through Balb/c mice immunization with HER2 overexpressing Calu3 cells (lung adenocarcinoma)^20^ and were reported to recognize HER2 ECD epitope I and epitope III, respectively^20,21^, while trastuzumab recognises epitope IV^14,22^.

Moreover, a complete characterisation of mAb-HER2 ECD interaction was performed by means of surface plasmon resonance (SPR): non-overlapping epitope was confirmed between MGR2 and MGR3 and between both antibodies and trastuzumab. Association and dissociation constants were calculated and trastuzumab was used as control mAb for both epitope mapping and kinetics analysis.

## Results

### HER2 ECD/mAbs epitope binding pattern evaluation

In this study, the binding pattern of anti-HER2 ECD mAbs was evaluated through a pair-wise binding approach, in which the ability of pairs of antibodies to bind simultaneously to the immobilised antigen was tested. The ability of multiple receptors to bind independently or to compete with each other for binding sites on a macromolecule can be easily tested by means of SPR, employing the Biacore technology^23–25^. With this technique, unlike conventional EIA (enzyme immunoassay) or RIA (radio immunoassay), there is no need for labelling of the reactants, and purification (cell supernatants can be used). Moreover, small amounts of reactants are sufficient for the epitope mapping^23^.

Concerning antibodies, those that are directed towards distinct epitopes will be able to bind simultaneously, while those that are directed towards the same epitopes will inevitably compete.

The monoclonal antibodies MGR2 and MGR3 were previously characterised and proved to recognise HER2 ECD epitope I and epitope III^20,21^, respectively, whereas trastuzumab recognises epitope IV^14,22^.

**Figure 1:**
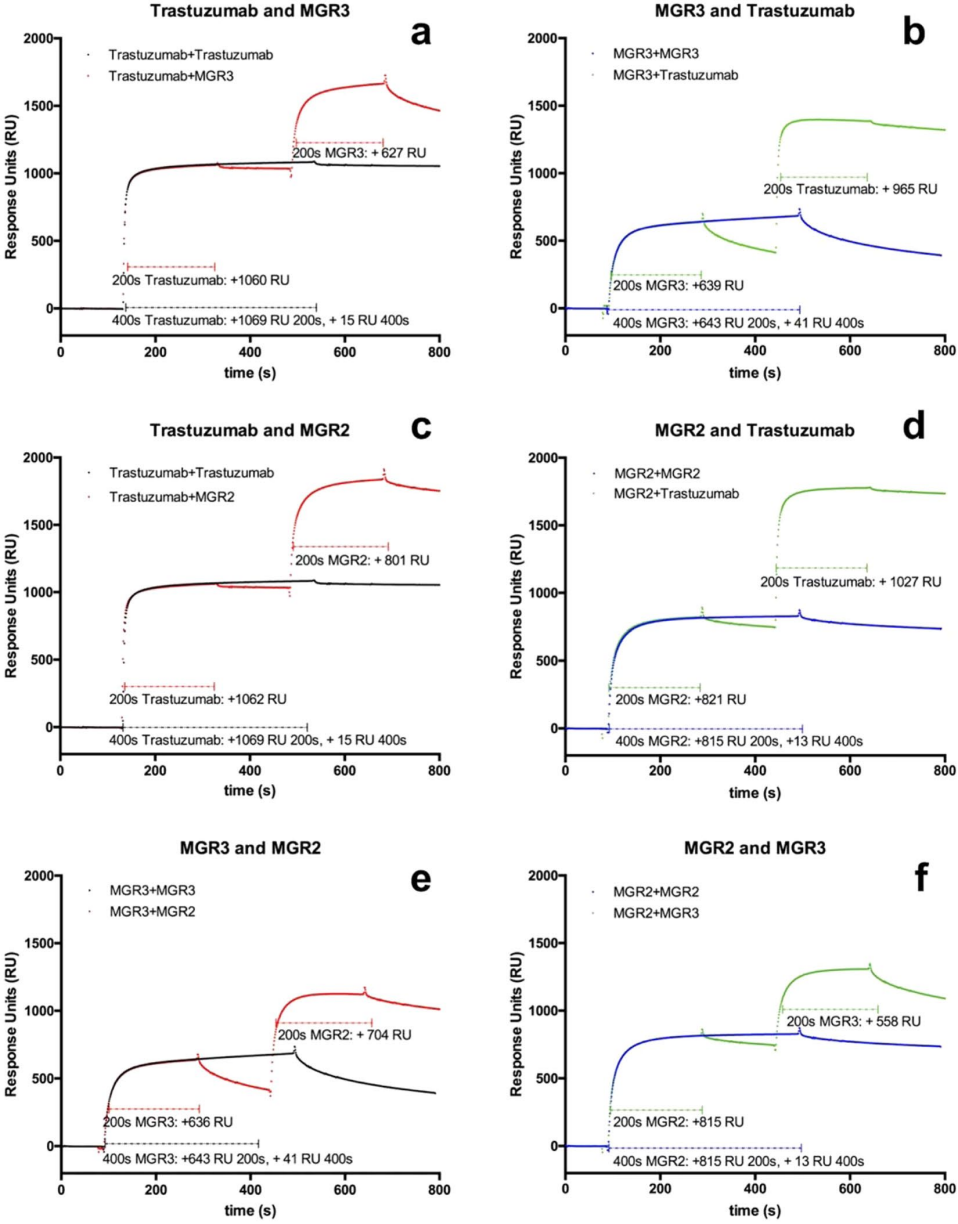
Competitive binding test for mAbs MGR2, MGR3, and trastuzumab toward immobilized HER2 ECD, determined by Biacore X100. (**a**) Trastuzumab (1st injection) vs MGR3 (2nd injection); (**b**) MGR3 (1st injection) vs trastuzumab (2nd injection); (**c**) trastuzumab (1st injection) vs MGR2 (2nd injection); (**d**) MGR2 (1st injection) vs trastuzumab (2nd injection); (**e**) MGR3 (1st injection) vs MGR2 (2nd injection) (**f**) MGR2 (1st injection) vs MGR3 (2nd injection).

HER2 ECD was covalently bound to the Biacore CM5 chip, and pairs of mAbs were sequentially injected following the pattern summarised in Table 1 and shown in Fig. 1, where the corresponding signals, expressed in response units (RUs), were recorded as sensograms after each injection.

**Table 1:** Anti HER2 ECD mAbs epitope binding pattern evaluation.

First Injection (RU)	Second Injection (RU)		
	+ MGR2	+ MGR3	+ trastuzumab
MGR2	815 + 13	815 + 558	821 + 1,027
MGR3	636 + 704	643 + 41	639 + 965
trastuzumab	1,062 + 801	1,060 + 627	1,069 + 15

It can be observed that the three mAbs do not compete for HER2 ECD binding. In fact, an increase of RU is observed when injecting the second mAbs (second injection) once the first one has reached saturation (first injection), thus confirming that epitopes recognised by MGR2, MGR3, and trastuzumab do not overlap. Comparable RU values were obtained switching mAbs in first and second injection, confirming that the binding of the first mAb does not interfere with the binding of the second one, independently from injection order. As for example, RU = 821 with MGR2 only, and an additional RU = 1,027 was gained when trastuzumab is added (Fig. 1d). When the injection order is reverted, a starting RU = 1,062 was reached with trastuzumab only, and an additional RU = 801 was gained when MGR2 was injected (Fig. 1c).

### mAbs - HER2 ECD SPR kinetics experiments

Several data are available in literature concerning the affinity constant (K_D_) determination for trastuzumab-HER2 ECD interaction using Biacore instruments. In general, all data refer to concentration ranging from sub-nanomolar to nanomolar.

In particular, K_D_ of 0.4 nM (k_a_ = 4.9 × 10^5^ M^−1^ s^−1^; k_d_ = 1.7 × 10^−4^ s^−1^)^26^, 1.9 nM (k_a_ = 1.9 × 10^6^ M^−1^ s^−1^; k_d_ = 3.4 × 10^−3^ s^−1^)^27^, 0.10 nM (k_a_ = 0.34 × 10^6^ M^−1^s^−1^; k_d_ = 0.42 × 10^−4^ s^−1^)^28^, 0.49 nM (k_a_ = 8.46 × 10^4^ M^−1^ s^−1^; k_d_ = 4.15 × 10^−5^ s^−1^)^29^, and 9.4 nM (k_a_ = 7.25 × 10^3^ M^−1^ s^−1^; k_d_ = 6.5 × 10^−5^ s^−1^)^30^ are reported, where K_D_ = k_d_/k_a_, while k_a_ and k_d_ are the kinetic constants for the association and dissociation events, respectively.

Concerning trastuzumab, the results obtained in this work were comparable with those reported in literature and mentioned above, which indicated a K_D_ in the sub-nanomolar range. Although some information were reported concerning MGR2 vs MGR3 *in vitro* effect^20^, their K_D_ was only recently published and showed quite relevant standard deviation values. On the other hand, no data were available concerning k_a_ and k_d_^31^.

In this work, we evaluated the binding properties of MGR2, MGR3 and trastuzumab toward HER2 ECD upon immobilisation of HER2 ECD onto CM5 chip.

Experiments were performed with mAbs diluted at the desired concentrations and injected over the functionalised CM5 chip surface (0.25–30 nM for trastuzumab, 1–75 nM for MGR2, 2.5–200 nM for MGR3).

Kinetics data showed K_D_ values of 0.19 nM (k_a_ = 6.09 × 10^5^ M^−1^ s^−1^; k_d = _1.15 × 10^−4^ s^−1^) for trastuzumab, 0.13 nM (k_a_ = 2.04 × 10^5^ M^−1^ s^−1^; k_d_ = 2.73 × 10^−5^ s^−1^) for MGR2 and 3.56 nM (k_a_ = 1.22 × 10^5^ M^−1^ s^−1^; k_d_ = 4.34 × 10^−4^ s^−1^) for MGR3.

**Figure 2:**
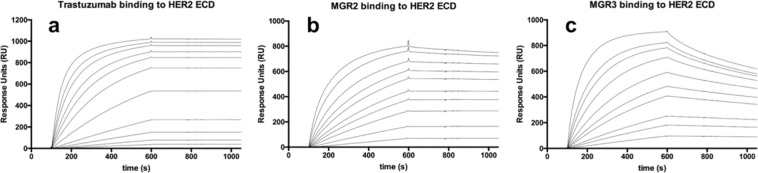
SPR sensograms showing the kinetic analysis performed on HER2 ECD antigen immobilized on CM5 chip. mAbs were tested at the following concentrations: (**a**) 0.25–30 nM for trastuzumab; (**b**) 1–75 nM for MGR2; (**c**) 2.5–200 nM for MGR3. Association time 500 s, dissociation time 400 s.

It is worth noting that K_D_ value for MGR2 showed the same order of magnitude than that for trastuzumab, while the K_D_ value for MGR3 is at least one order of magnitude higher, mainly due to a higher k_d_. This finding can be easily visualised in the sensograms shown in Fig. 2, where it is possible to appreciate a difference between the dissociation phase of MGR3 (Fig. 2c, estimated k_d_ = 4.34 × 10^−4^ s^−1^) when compared to the dissociation phase of trastuzumab (Fig. 2a, estimated k_d_ = 1.15 × 10^−4^ s^−1^) and of MGR2 (Fig. 2b, estimated k_d_ = 2.73 × 10^−5^ s^−1^). With respect to the K_D,_ one order of magnitude difference between MGR2 and MGR3 was also previously reported^31^.

A comparison of the kinetic data for MGR2 and MGR3 shows that the former mAb has higher affinity for HER2 ECD in terms of both association and dissociation. However, the SPR data demonstrate the suitability of both the selected mAbs (MGR2 and MGR3) to be tested as capture antibodies for ELISA, as it will be discussed in the following paragraphs.

### Development of sandwich ELISA procedure with mock samples

A sandwich ELISA protocol was optimised step-by-step by employing mock samples prepared by spiking the buffer with different concentrations of recombinant HER2 ECD protein as the antigen, MGR2 or MGR3 as the capture antibodies, and a rabbit anti-human polyclonal antibody as the detection antibody. Serial dilutions of capture and detection antibodies were used to determine the working concentration for each reagent. The selected concentrations were 2 µg mL^−1^ and 5 µg mL^−1^ for the capture antibodies MGR2 and MGR3, respectively, and 0.7 µg mL^−1^ for the detection antibody.

#### Optimization of blocking agent and time

The blocking step in ELISA is a key factor to reduce non-specific binding^32^, which can result in a high background signal, and poor sensitivity and specificity. Two commonly used protein blockers^33^ were selected and evaluated as blocking agents to reduce non-specific binding to unoccupied sites on the surface of ELISA plates: bovine serum albumin (BSA) and human serum albumin (HSA). HSA was preferred over BSA as it provided lower backgrounds and higher recovery performances. Thus, 2% HSA in PBS was used in all subsequent experiments as the blocking buffer. Samples and detection antibodies were prepared in a buffer containing a lower HSA concentration (1%) plus 0.1% Tween-20 in order to further reduce the background and minimize non-specific hydrophobic interactions^34^.

#### ELISA analytical validation

**Figure 3:**
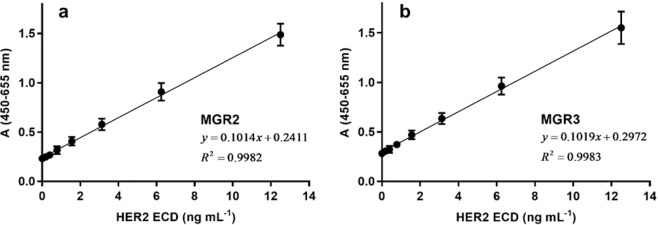
ELISA standard curve for HER2 ECD quantification in the range 0–12.5 ng mL^−1^, employing (**a**) 2 µg mL^−1^ MGR2 or (**b**) 5 µg mL^−1^ MGR3 mAbs as the capture antibodies and anti-HER2 ECD pAb as detection antibody. Error bars indicate standard deviation (SD).

The sandwich ELISA was developed to detect HER2 ECD protein using MGR2 or MGR3 and a rabbit anti-HER2 ECD polyclonal antibody as capture and detection antibodies, respectively. We addressed several typical steps and optimised the parameters required for validation analysis to evaluate actual feasibility and possible challenges. Calibration curves for HER2 ECD recombinant protein were obtained in the concentration range from 0.19 to 12.50 ng mL^−1^ with both MGR2 and MGR3 as capture antibodies. Both assays provided a good linearity of the dose-response signal (Fig. 3) with correlation coefficients greater than 0.99.

Coefficients of *intra*- and *inter*-assay variation were assessed among replicates of at least twelve assays performed in different days under the same operating conditions^35^, and resulted lower than 10% and 15%, respectively (Supplementary Table S1), thus suggesting good assay precision. The lower limit of detection (LOD) and the lower limit of quantification (LOQ) were calculated as the concentrations corresponding to the mean absorbance of the zero plus three or ten times its standard deviation, respectively^35^. LODs were determined to be 0.76 ng mL^−1^ for MGR2 and 0.75 ng mL^−1^ for MGR3. Similarly, the LOQs were 2.77 ng mL^−1^ and 2.85 ng mL^−1^ for MGR2 and MGR3, respectively (Supplementary Table S1).

Quantification of HER2 ECD was performed by analysing two-fold serial dilutions of recombinant protein in assay buffer. The observed protein concentrations were in agreement with the expected values with a deviation of ±20%, thus indicating assay linearity. The theoretical concentration was used to calculate percentage of recovery, which proved the assay reliability, as for it was included in the ranges of 85.4–115% (mean 102.2%) and 91.2–106.6% (mean 98%) for MGR2 and MGR3-based assays, respectively.

#### ELISA mAb – pAb optimization and developing of dual mAbs-based sandwich ELISA

To improve signal amplification, the ELISA protocol was optimized using the biotin-streptavidin conjugation strategy. In fact, the use of the high-affinity and non-covalent interaction between biotin and streptavidin produced a maximized signal-to-noise with increased sensitivity compared to conventional enzyme-labeled secondary antibody^36^. Considering the high affinity shown in SPR experiments, MGR2 was selected as capture antibody and a goat anti-HER2 ECD biotinylated polyclonal as detection antibody. The calibration curve showed a linear behaviour in the range of 0.19–12.50 ng mL^−1^ with a correlation coefficient of 0.998.

**Figure 4:**
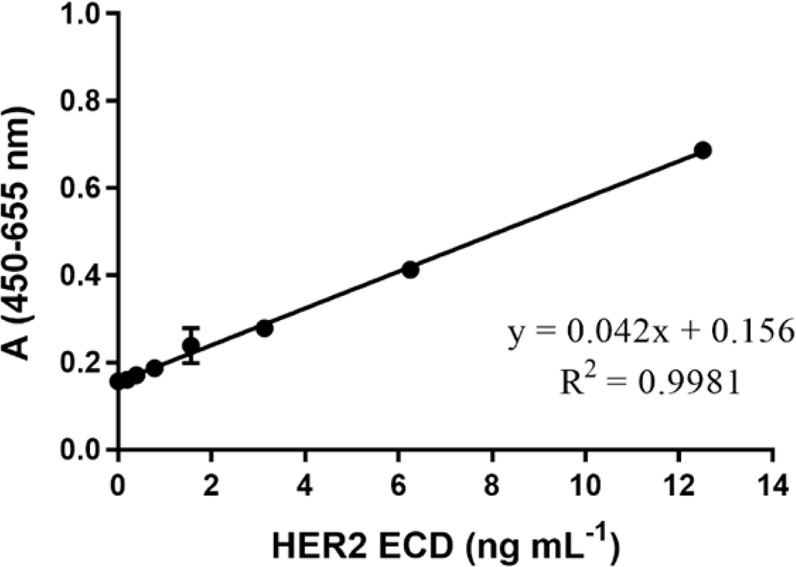
ELISA standard curve for HER2 ECD quantification in the range 0.19–12.5 ng mL^−1^, employing 5 µg mL^−1^ MGR3 as the capture antibody and 15 µg mL^−1^ biotinylated MGR2 as detector mAb. Error bars indicate SD.

Since MGR2 and MGR3 recognize two non-overlapping epitopes in HER2 ECD, a sandwich ELISA based on these two mAbs was also developed. To exploit the biotin-streptavidin conjugation strategy, the biotinylation of MGR2 and MGR3 was performed using the EZ-Link™Sulfo-NHS-LC Biotinylation Kit (code 21435, Thermo Fisher Scientific) following manufacturer’s instructions. MGR2 and MGR3 (1 mg mL^−1^) were labelled with 6.60 and 5.45 molecules of biotin, respectively. Assay set-up using MGR3 as capture antibody (5 µg mL^−1^) and biotinylated MGR2 as detection (15 µg mL^−1^) was identified as the best configuration in terms of curve range (0.19–12.50 ng mL^−1^) and coefficient of determination R^2^ (0.998) (Fig. 4).

Although the use of the dual mAb sandwich ELISA allowed also an acceptable detection in assay buffer, the pAb-based strategy was herein preferred for detection of native HER2 ECD in serum samples. In fact, native proteins in biological matrices like serum are more complex and their conformation could be altered by several factors affecting the strength of mAb-antigen interaction, including post-translational changes, temperature, pH range and salt concentration. These aspects are of minor concern when employing pAbs, which are more stable to pH and salt concentration variations. Moreover, the ability of pAbs to bind multiple epitopes allows signal amplification in sample with low level of expression of the target protein^37^.

#### ELISA application to HER2 ECD spiked serum samples

Assay performance when quantifying HER2 ECD spiked in serum samples was evaluated in 11 serum matrix using samples from healthy donors available in the biobank of the Regional Centre Biomarkers and already tested as negative for soluble HER2 ECD (ADVIA Centaur HER2/neu assay – Ref n° 00915031, Siemens Healthcare Diagnostics)^38^.

The potential effect of interferences arising from serum matrix was overcome by performing a 10-fold dilution of serum in the experimental buffer and by choosing HSA as blocking agent, which led to reduced backgrounds and higher recovery performances. In this setting, the analysis of HER2 ECD negative sera provided absorbance values comparable to the signal obtained with the experimental buffer alone, thus revealing an absence of matrix interference with respect to the quantification of HER2 ECD biomarker and reflecting the specificity of the test.

Assays performances for quantification of fixed amounts of recombinant HER2 ECD spiked in human serum, diluted 1:10 in the experimental buffer, was compared to the quantification of similar amounts of protein in experimental buffer. Assay accuracy and specificity were confirmed under these conditions, resulting in proper analytes quantification even in presence of potentially interfering serum components.

In particular, MGR2 provided a percentage of recovery of 100.8% (range 94–119.9%) in human serum spiked with HER2 ECD, while MGR3 performed slightly worse in human serum with a recovery of 76.8% (range 65.3–92.7%). This evidence agreed well with the kinetic data acquired with the Biacore for MGR2 and MGR3, which showed that the former mAb has higher affinity for HER2 ECD in terms of both association and dissociation constants.

On these grounds, the assay relying on MGR2 and biotinylated pAb as capture and detection antibodies was selected for the validation of HER2 ECD measurements in clinical samples.

#### ELISA validation for HER2 ECD measurements in clinical samples

The validation process included the determination of precision, LOD, linearity, method comparison and bias estimation using patient samples. For all tests, samples were previously diluted 1:10 in assay buffer and tested in duplicate.

The assay was evaluated using serum samples of patients with histological diagnosis of stage II/III primary breast cancer (BC) collected between 1998 and 2001 and available in the biobank of the Regional Centre Biomarkers.

Coefficients of *intra*- and *inter*-assay variation were assessed as previously described and resulted lower than 11 and 15%, respectively, suggesting an acceptable assay precision for the HER2 ECD measurement in clinical samples. LOD and LOQ were 0.76 ng mL^−1^ and 2.77 ng mL^−1^, respectively. Both assays showed high sensitivity, proving their suitability to detect HER2 ECD when present at low concentration in clinical samples.

The linearity of quantitative measurement procedure of HER2 ECD in clinical samples was determined accordingly to the EP06-A guideline^39^. Two HER2 ECD positive serum samples were selected from the biobank: the first one, containing 6.1 ng mL^−1^ of HER2 ECD, namely ‘serum low’ (SL), while the second one containing 8.7 ng mL^−1^, namely ‘serum high’ (SH). Five samples were prepared with different SL:SH ratios: 1:0 (sample 1); 3:1 (sample 2); 1:1 (sample 3); 1:3 (sample 4); and 0:1 (sample 5). Results are reported in Table 2 and Supplementary Fig. S1 shows the linearity of the measures.

**Table 2:** Linearity of quantitative measurement procedure represented as percentage recovery (Expected/Observed HER2 ECD level) for different proportions SL (Serum Low) and SH (Serum High).

Sample	SL:SH ratio	Expected HER2 ECD level (ng mL^−1^)	Observed HER2 ECD level (ng mL^−1^)	% Recovery
1	1:0	6.1	6.06	99.5
2	3:1	6.8	6.2	91.3
3	1:1	7.3	6.7	91.6
4	1:3	8	7.9	98.3
5	0:1	8.7	8.6	99.9

Moreover, a comparison with two established commercial tests, used as reference, was conducted employing 22 serum samples for the agreement analysis (11 for each method). The serum HER2 ECD levels previously tested with commercial methods (ADVIA Centaur HER2/neu assay, Siemens Healthcare Diagnostics – Ref n° 00915031 and Quantikine Human ErbB2/HER2 Immunoassay, No. DHER20, R&D Systems) ranged from 3.6 to 26.7 ng mL^−1^ (Supplementary Table S2).

**Figure 5:**
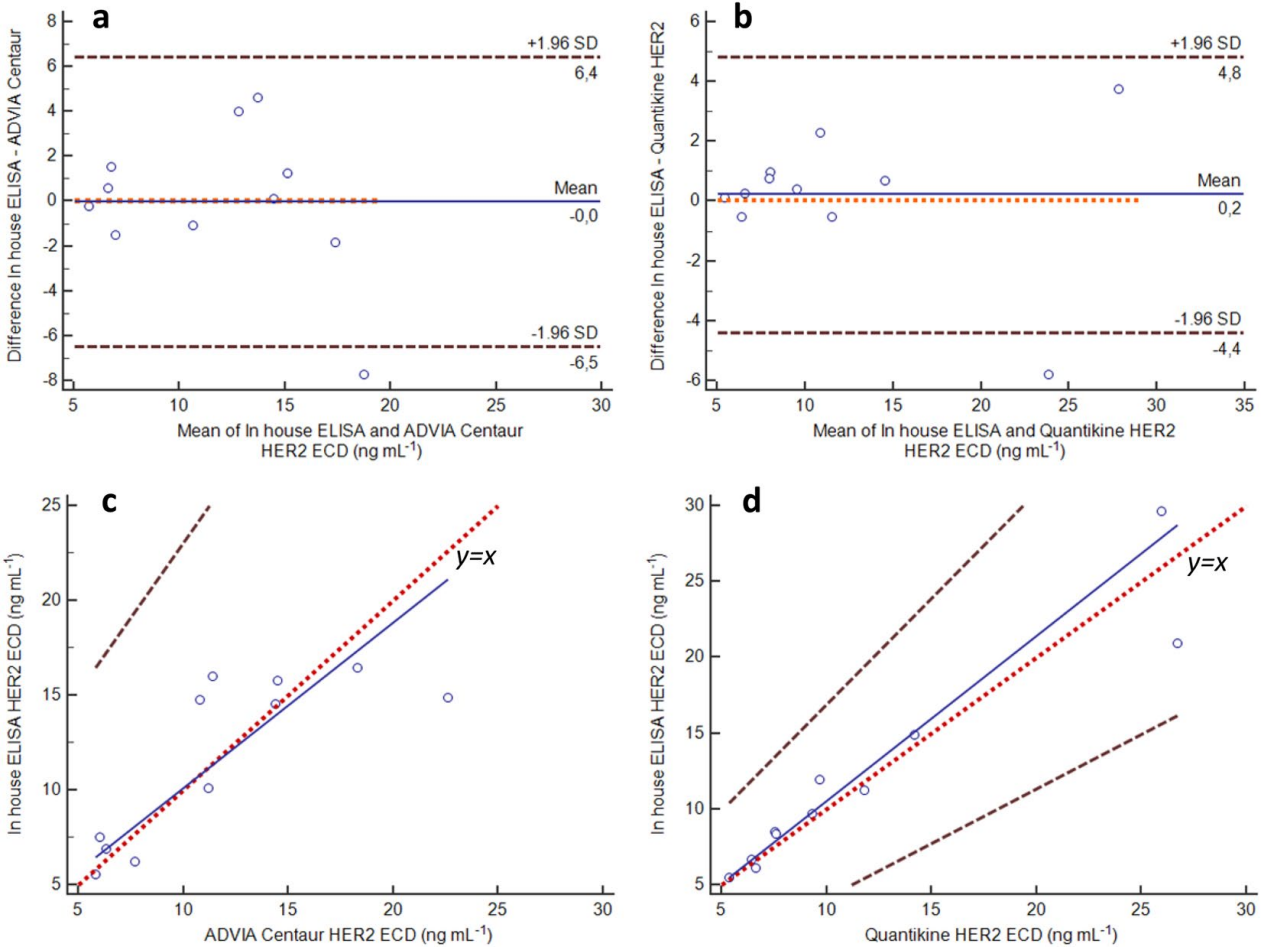
Bland-Altman plot of differences between (**a**) ADVIA Centaur or (**b**) Quantikine HER2 ELISA kit and *in house* developed ELISA. Passing-Bablok regression analysis (n = 11 serum samples) comparing (**c**) ADVIA Centaur or (**d**) Quantikine HER2 ELISA kit with the *in-house* ELISA. In Bland-Altman analysis (panels a and b), the difference between the tested methods is plotted against the mean for HER2 ECD level. Black dotted lines show limits of agreement (95% CI) and the blue line shows the mean value of the differences between the two methods under comparison (the bias). The red dotted line is the zero line for assessing the discrepancy of the observed mean difference from zero. In Passing–Bablok agreement analyses (panels c and d), the data obtained with the *in-house* ELISA (y axis) is plotted against those of the commercial reference methods (x axis) together with the identity line (y = x) and the regression line. The regression line is continuous and blue, the red dotted line is the identity line (y = x) and the black dashed lines are the CI. Results are summarised as it follows. (**c**) regression equation, y = 1.41 + 0.87x; intercept, 1.41 (95% CI −5.85 to 7.39); slope, 0.87 (95% CI 0.33 to 1.56). (**d**) regression equation, y = −0.37 + 1.08x; intercept, −0.37 (95% CI −3.17 to 2.86); slope, 1.08 (95% CI 0.72 to 1.40).

Bland-Altman plots and Passing-Bablok regression analyses were used to assess agreement between the methods using MedCalc (v 14.10.2). The 95% upper and lower confidence intervals (CI) were estimated at −6.5 and 6.4 when the developed assay was compared with ADVIA Centaur. The mean difference in the plot, due to the bias, was equal to zero, thus indicating that HER2 ECD measurements with the *in-house* developed ELISA are in agreement with those obtained with ADVIA Centaur (Fig. 5a). Similarly, the agreement analysis between *in-house* ELISA and the commercial ELISA kit Quantikine HER2 (Fig. 5b) showed a distribution of HER2 ECD values within the limits of agreement (95% CI, −4.4 and 4.8). In this case, the bias was close to zero (mean difference of 0.2). Moreover, in both comparative analyses using Passing-Bablok regression (Fig. 5c,d), the 95% CI for the intercept included the value 0, while the 95% CI for the slope included the value 1. Therefore, it can be stated that the developed assay and the two reference methods are in very good agreement, with an average bias in the range from 0 to +0.2 as shown before (Fig. 5a,b).

It is worth to emphasize that agreement analysis provided also information about the *ex-vivo* stability of HER2 over time. In fact, the agreement between HER2 ECD levels assessed with the developed assay and those analysed with ADVIA Centaur in samples collected between 1998 and 2001 demonstrated that HER2 ECD levels in samples stored in a bank for about 20 years at −80 °C are comparable with those measured in fresh samples. These results agree very well with previous reports describing no significant changes in the HER2 ECD levels after sample collection and storage at room temperature as well as after refrigeration at 2–8 °C or freezing at −20 or −80 °C^40–42^.

#### Evaluation of trastuzumab interference

As previously confirmed by SPR experiments, epitopes that are recognized by MGR2, MGR3 and trastuzumab do not overlap.

The competitive binding of MGR2, MGR3, and trastuzumab towards HER2 ECD was also investigated by displacement tests. Before starting the experiment, HER2 ECD solutions were pre-incubated with two concentrations of trastuzumab (50 and 200 µg mL^−1^), which are included in the range of concentration within the mean minimum and the mean maximum measured in serum of patients, corresponding to 66 and 110 mg L^−1^, respectively^40^. The solutions were then assayed with the MGR2 or MGR3-based assays and compared with freshly prepared HER2 ECD solutions.

**Figure 6:**
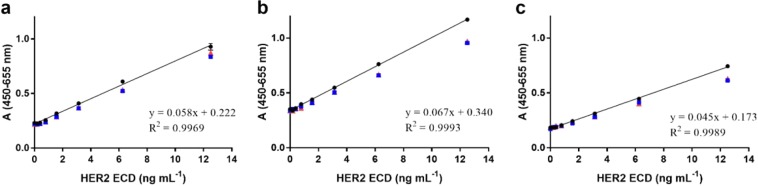
Determination of HER2 ECD only (black dots) or pre-incubated with trastuzumab at 50 (red triangles) and 200 (blue squares) µg mL^−1^ employing (**a**) MGR2- or (**b**) MGR3-based assay and (**c**) the dual mAbs sandwich ELISA with MGR3 in coating and biotinylated MGR2 as the detection antibody. Error bars indicate SD but are too small to be visible.

Noteworthy, the interference was limited and independent from the concentration of trastuzumab, thus suggesting a possible steric hindrance instead of a competition for HER2 ECD binding. Trastuzumab interference was higher when HER2 ECD was at very low concentrations (3.13–1.56 ng mL^−1^), close to the LOQ of the ELISAs (2.77 or 2.85 ng mL^−1^ of HER2 ECD). Pre-incubation with 50 µg mL^−1^ of trastuzumab led the investigated HER2 ECD concentration of 12.5 ng mL^−1^ to decrease of 7.9% or 23.9% with the MGR2- or MGR3-based assay, respectively (Fig. 6a,b).

We hypothesize that this phenomenon might be due to the stoichiometric excess of the drug towards HER2 ECD binding-site during the pre-incubation phase. It could be also observed that MGR3 was affected by trastuzumab to a higher extent, when compared to MGR2, probably because of its lower affinity for HER2 ECD, as witnessed by SPR experiments.

Trying to overcome the hypothetical steric hindrance due to the binding of pAb to more than one epitope, and to balance the stoichiometry between the antibodies used, we performed a displacement test using the dual mAbs sandwich ELISA, with the configuration described previously. The experimental set up for trastuzumab displacement was maintained, testing 50 and 200 µg mL^−1^ of drug. Pre-incubation with 50 and 200 µg mL^−1^ of trastuzumab lowered the detection limit of 12.5 ng mL^−1^ of HER2 ECD of 19.5 and 22.5%, respectively. However, the interference decreased at lower concentrations of HER2 ECD (0.78 and 1.56 ng mL^−1^) with percentages ranging from 4.66 to 15.5% while, at 0.39 ng mL^−1^ near the LOD (0.10 ng mL^−1^) and LOQ (0.30 ng mL^−1^) the interference is absent (Fig. 6c).

To evaluate the presence of a significant difference between the linear regression curves of samples pre-incubated with trastuzumab and the standard curve, the analysis of covariance (ANCOVA) was performed. Neither the values of every slope, nor the related intercepts differed significantly from the standard solutions, indicating that the presence of trastuzumab in both the tested concentrations did not affect significantly the measured levels of HER2 ECD. Moreover, adjusting the concentration of the detection antibody allowed a reduction of the interference at lower HER2 ECD concentration with an optimization between the stoichiometric ratio of the antibodies involved in the detection.

## Discussion

This study describes the development of a sandwich ELISA based on epitope specific monoclonal antibodies for HER2 ECD quantification. As previously described, monoclonal antibodies MGR2 and MGR3 recognise HER2 ECD epitope I and epitope III, respectively^20,21^. Moreover, they do not compete for the same binding site of trastuzumab, thus allowing to quantify soluble HER2 ECD, even when linked to trastuzumab.

The characterisation of epitope specificity patterns with a panel of mAbs gives valuable information to employ mAb in clinical, diagnostic, and technical contexts^23^. In this respect, the use of MGR2 or MGR3, as appropriately selected capture mAbs that recognise specific and characterized epitopes, would have the potential to allow correlations of HER2 ECD with its clinical role qualitatively, as well as quantitatively.

Cancer investigators currently face challenges with respect to mAbs. In fact, the majority of antibodies are poorly characterized and not adequately validated for the variety of applications of interest to the research community^41^. As discussed by Haab *et al*.^42^, the user is forced to orient her/himself in an increasingly complex and intricate marketplace to determine whether data are available concerning the binding characteristics of an antibody and whether the antibody is suitable for a specific application.

Smaller in-house production operations can offer better characterizations of the antibodies than those offered by big companies^43^. In this context, although antibody-based assays exist, which employ antibodies that bind to different epitopes and that do not compete with trastuzumab^44^, the antibodies used in commercial ELISA kits do not recognise specifically mapped epitopes on HER2 ECD molecules. In this work, we exploited MGR2 and MGR3 mAbs that recognise and bind to specific epitopes of HER2 ECD to develop a sandwich ELISA. Our findings showed, for the first time in this particular setting, lack of competition between MGR2 or MGR3 and trastuzumab, which specifically binds on the C-terminal portion of HER2 epitope IV. Presumably these mAbs do not compete also with the epitope II, which instead is recognized by pertuzumab^45^. Furthermore, our results demonstrated the possibility to use both mAbs in the same assay as capture and detector, reaching good specificity and sensitivity also at low HER2 ECD levels and in presence of trastuzumab with no interference for quantifying the target protein. Moreover, the performance of this newly designed assay was compared against two validated commercial tests in quantifying HER2 ECD in serum samples and a good agreement among the three methods was observed in the majority of clinical samples, suggesting that the three methods are equally competent in identifying HER2 ECD, even at a low protein level.

Antibodies currently used in commercial ELISA kits do not recognise specifically mapped epitopes on the HER2 ECD protein. In this work, we investigated the properties of two highly characterized mAbs, MGR2 and MGR3, to develop and propose a reliable ELISA method to detect, quantify, and monitor HER2 ECD levels at concentrations that lay below the working range of commercial assays. Our findings also showed that such epitope recognition is not interfering with the use of anticancer drug trastuzumab, thus making the developed ELISA assay a promising tool to monitor HER2 ECD levels also in patients under trastuzumab treatment. Moreover, the epitope-specificity of MGR2 and MGR3 antibodies increase their potential applicability in the development of new analytical tools that can be employed in the clinical practice. To conclude, we strongly believe that the present strategy shows promising applicability to be optimized in an assay for biomarkers quantification, as for example those describing the multiplexed electrochemical detection of protein biomarkers^46^ with the aim of establishing a point-of-care testing (PoCT) approach.

## Methods

### Chemicals

All the reagents and buffer components were purchased from Sigma-Aldrich, unless otherwise specified. Bi-distilled (dd-H_2_O) or Milli-Q grade water was used. Mouse monoclonal antibodies (IgG mAbs) MGR2 and MGR3 directed against the extracellular domain of HER2, obtained and purified as previously described^20^ were stored sterile at 4 °C in a buffer containing 150 mM sodium chloride and 0.02% sodium azide at pH 7.4.

Recombinant HER2 ECD protein (code 10004-HCCH, 70 kDa), and rabbit anti-human HER2 polyclonal antibody (IgG pAb, code 10004-RP04) were obtained from Sino Biological Inc. and stored in multiple aliquots at −20 °C. Goat anti human HER2 biotinylated polyclonal antibody (IgG pAb-biotin, code BAF1129) and Streptavidin-HRP conjugate (code DY998 Part #890803 5 vials) were from R&D Systems and stored at −20 °C and at 4 °C respectively. Goat anti-rabbit IgG-HRP conjugate (1 mg mL^−1^, code 401353) and 3,3′,5,5′-Tetramethylbenzidine (TMB) liquid substrate system for ELISA (code ES001) were purchased from Merck Millipore and stored at 4 °C together with lyophilized human serum albumin (HSA, code A1653, Sigma-Aldrich).

Herceptin (trastuzumab 150 mg, Roche) was kindly provided by Dr. Renzo Lazzarini, Pharmaceutical Division, CRO Aviano, resuspended and stored as recommended by the manufacturer.

### SPR sensor chip preparation and interaction assays

SPR measurements were performed with Biacore X100 instrument (GE), at 25 °C, with a flow rate of 30 µL min^−1^. CM5 sensor chips, running buffer (HBS-EP+) and Amine Coupling Kit (1-ethyl-3-(3-dimethylaminopropyl) carbodiimide hydrochloride (EDC), N-hydroxysuccinimide (NHS), 1.0 M ethanolamine-HCl pH 8.5) were purchased from GE.

The binding properties of MGR2, MGR3 and trastuzumab to HER2 ECD were evaluated upon immobilization of HER2 ECD onto CM5 chip surface in flow cell 2 (FC-2), using Biacore Amine Coupling Kit according to the manufacturer’s instructions. HER2 ECD was preliminary diluted to 10 µg mL^−1^ in acetate buffer (pH 4.5) and ≈1,000 RU (Response Units) of HER2 ECD were immobilized onto the chip surface. Blank amine immobilization was performed on flow cell 1 (FC-1), used as reference.

Epitope overlapping/competition experiments and kinetics experiments were performed with mAbs diluted in HBS-EP + buffer 1X at the desired concentrations and injected over the functionalized surface. For kinetics experiments, 10 to 12 dilutions in the low nanomolar range were tested for each mAb.

Epitope overlapping/competition experiment were performed through a pair-wise binding approach by injecting one mAbs on HER2 EDC functionalised chip until binding sites saturation is reached (200 s), followed by a second injection (200 s) of the second mAb to evaluate competition.

Surface regeneration was performed with NaOH 10 mM for 30 s for both MGR2 and MGR3, and with NaOH 50 mM for 20 s for trastuzumab. Surface regeneration conditions were evaluated and established after appropriate pH scouting experiments.

All sensograms were corrected by subtracting the signal recorded on FC-2 (functionalised cell) from the signal recorded of FC-1 (reference cell); collected data were evaluated by non-linear analysis of the association and dissociation curves using SPR kinetic evaluation software (BIAevaluation Software, version 2.0, Biacore). Data fitting was carried out with a 1:1 Langmuir binding model to obtain k_a_, k_d_ and K_D_ = k_d_/k_a_.

Fitting analysis was evaluated for all the performed assays and was compliant to the following statistical parameters: χ^2^ < 5% of R_max_, U-value < 25, SE (standard error) values < 5% of the corresponding parameters and tc > 10^8^ (tc: flow rate independent part of the mass transfer constant).

### ELISA procedure

96-well microtiter plates (code 655001, Greiner Bio-One) were coated with mAb MGR2 or MGR3 by incubating overnight at 4 °C with 100 µL/well of antibody at 2 µg mL^−1^ or 5 µg mL^−1^ in 10 mM of phosphate buffer pH 7.4 (PBS, code 524650, Merck Millipore), respectively. After removal of the coating solution, the coated plates were blocked with 300 µL/well of PBS containing 2% HSA for 1 h at 37 °C. Recombinant HER2 protein was diluted at 12.5 ng mL^−1^ in PBS containing 1% HSA and 0.1% Tween 20 (experimental buffer) and used to generate a serial two-fold dilution standard curve. Standards and samples were dispensed onto the coated wells (100 µL/well), and plates were incubated at room temperature for 1 h. Rabbit pAb (0.7 µg/mL in experimental buffer) was then added (100 µL/well) and plates were incubated at room temperature for 1 h. Horseradish peroxidase (HRP)-conjugated goat anti-rabbit IgG was then used as second labelled antibody (diluted 1:5,000 in experimental buffer, 100 µL/well). After further incubation for 30 min at room temperature, TMB substrate solution was added to develop the plates (100 µL/well) and the colour reaction was blocked after 10 min incubation at room temperature by addition of 0.18 M sulfuric acid (100 µL/well). Absorbance values at 450 nm and 655 nm were determined on a Biorad iMark microplate reader. When using the biotinylated pAb (0.2 µg mL^−1^ in experimental buffer) or the biotinylated mAbs (15 µg mL^−1^ in experimental buffer) as detector antibody the following incubation was made using the Streptavidin-HRP conjugate (diluted 1:200 in experimental buffer, 100 µL/well). After each incubation step, plates were washed three times with PBS containing 0.1% Tween 20 (wash buffer) using an automated wash station (Bioplex Pro II) to remove unbound antigen and/or antibody. SigmaPlot 12.5 software (Systat Software, Inc.) was used to determine the concentration of recombinant HER2 ECD protein from absorbance values by using a recombinant HER2 ECD protein dissolved in experimental buffer at known concentrations as reference. Calibration curves were interpolated with a linear regression model. Linearity of the analytical procedure in the identified working range was evaluated by linear regression among data points.

### Serum samples and ethical compliance

The overall study was conducted in accordance to the Declaration of Helsinki and its revisions and approved by the Local Ethical Committee (protocol 77190/2016, Ethics Committee for Clinical Experimentation of the Province of Venice and IRCSS San Camillo), which is organized and operates in accordance with Good Clinical Practice and the applicable laws and regulations. All tests were performed in accordance with relevant guidelines and regulations. A total of 33 serum samples obtained from the biobank of the Regional Centre for Biomarkers were tested in this study. HER2 ECD negative samples (n = 11), used to assess the precision and the linearity of the *in-house* ELISA, were obtained from healthy subjects who gave the informed consent for the use of samples for biomedical research in the field of tumor biomarkers at the time of donation to the biobank. HER2 ECD positive samples (n = 22), used to compare the newly designed assay with the two commercial tests, were obtained from patients with primary BC previously collected for diagnostic purposes, whose data were anonymized by coding. For these samples archived for more than twenty years, the need for informed consent was waived by the ethics committee in accordance with the requirements of Italian law (Italian Data Protection Authority - Garante Privacy, Authorisation no. 9/2014 – General Authorisation to Process Personal Data for Scientific Research Purposes. Published in Italy’s Official Journal No. 301 on 30^th^ December 2014). After collection, all clinical samples were properly stored at −80 °C until quantification.

### EZ-Link™Sulfo-NHS-LC biotinylation Kit

The biotinylation kit (code 21435, Thermo Fisher Scientific) includes desalting columns for purifying the labelled antibody and 4′-hydroxyazobenzene-2-carboxylic acid (HABA) and avidin for measuring biotin incorporation. The biotinylation of MGR2 and MGR3 was performed following manufacturer’s instructions using a 20-fold molar excess of NHS ester-activated biotin to label 1 mg mL^−1^ of mAbs to attach 4–6 biotin groups per molecule. Briefly, 1 mg mL^−1^ of MGR2 or MGR3 was dissolved in 10 mM PBS provided. 6.8 µL of 10 mM Sulfo-NHS-LC Biotin, previously dissolved in ultrapure water, were added to both mAbs to react and incubated on ice for two hours. To remove the excess biotin reagent, the desalting columns were used after placing them into a 15 mL tube and centrifuging at 1000 g for 2 min. The columns were then equilibrated for three times by adding 2.5 mL of PBS to the top of the resin and centrifuging at 1000 g for 2 min. The solution containing the biotinylated mAbs was spotted in the centre of the resin bed to allow the sample to absorb. A final centrifugation step was made to collect the purified labelled mAb. To estimate biotin incorporation, the HABA assay was performed by pipetting 180 µL of HABA/avidin solution into a micro well of a 96-well plate; the absorbance at 500 nm was measured and recorded as reference A_500 nm_ HABA/Avidin; 20 µL of biotinylated mAb was added to the well and mixed; the absorbance at 500 nm was re-measured and biotin, thanks to its high affinity for biotin, displaces HABA interacting with avidin, and the absorbance at 500 nm decreases proportionately. According to the kit calculation procedures, 6.60 and 5.45 molecules of biotin were incorporated to MGR2 and MGR3, respectively.

### Evaluation of serum HER2 ECD with ADVIA Centaur HER2 assay

HER2 ECD measurements in serum samples were performed as described before^38^ using this fully automated two-site sandwich immunoassay based on two specific anti-HER2 ECD mAbs (TA-1 and NB-3) and direct chemiluminescent technology, accordingly to manufacturer ‘s instructions. *Intra*- and *inter*-assay CV% were less than 5%.

### Evaluation serum HER2 ECD with Quantikine Human ErbB2/HER2 immunoassay

This commercial kit (code DHER20, R&D Systems), which was used according to manufacturer ‘s instructions, is a quantitative sandwich ELISA assay based on an anti-HER2 mAb pre-coated onto a microplate. *Intra*- and *inter*-assay CV% were less than 4%.

### Evaluation of trastuzumab interference

A solution was prepared diluting trastuzumab at 4 mg mL^−1^ in NaCl 9 g L^−1^ and stored in the dark at 4 °C. This solution was used as stock solution. Two concentrations of trastuzumab as interfering agent (50 or 200 µg mL^−1^) were added to the prepared HER2 test solutions, and the observed values were compared to those from standard HER2 calibration curve. In brief, before starting the experiment, the HER2 ECD test solutions were pre-incubated with 0 (only experimental buffer), 50 or 200 µg mL^−1^ of trastuzumab for 30 min at 4 °C to cover the concentration range 66–110 µg mL^−1^, found in serum samples by pharmacokinetics studies^36^. The three pre-incubated standard curves were then assayed and compared on the developed ELISA assays.

## Supplementary information

Supplementary Materials.

## Data Availability

The authors declare that all data supporting the findings of this study are available within the paper and its supplementary information files.
