# Supplementary material for: ELISA assay employing epitope-specific monoclonal antibodies to quantify circulating HER2 with potential application in monitoring cancer patients undergoing therapy with trastuzumab
Source: Sci Rep. 2020 Feb 20;10:3016. doi: 10.1038/s41598-020-59630-y (PMC7033231; doi:10.1038/s41598-020-59630-y)

Supplementary Material

ELISA assay employing epitope-specific monoclonal antibodies to quantify circulating HER2 with potential application in monitoring cancer patients undergoing therapy with trastuzumab

Valentina Agnolon[1,+], Anna Contato[1,+], Anna Meneghello[2,+], Elda Tagliabue[3], Giuseppe Toffoli[2], Massimo Gion[1], Federico Polo[2,*], Aline S. C. Fabricio[1,*]

[1]Regional Center for Biomarkers, Department of Clinical Pathology and Transfusion Medicine, Azienda ULSS 3 Serenissima, Regional Hospital, Campo SS Giovanni e Paolo 6777, 30122 Venice (VE), Italy

[2]Experimental and Clinical Pharmacology, Centro di Riferimento Oncologico (CRO) IRCCS, Via F. Gallini 2, 33081 Aviano (PN), Italy

[3]Molecular Targeting Unit, Fondazione IRCCS Istituto Nazionale dei Tumori, Via Venezian 1, 20133 Milan (MI), Italy

[*]federico.polo@unive.it

[*]aline.fabricio@aulss3.veneto.it

[+]These authors contributed equally to this work and are listed in alphabetical order

V. A. present address: Division of Immunology and Allergy, Centre Hospitalier Universitaire Vaudois (CHUV), Rue du Bugnon 46, 1011 Lausanne, Switzerland.

A.M. and F.P. present address: Department of Molecular Sciences and Nanosystems, Ca' Foscari University of Venice, Via Torino 155, 30172 Venezia, Italy

Supplementary Table S1. Assay repeatability, LOD and LOQ for MGR2- and MGR3-based assays in mock samples.

	MGR2	MGR3
% CV intra-assay	0.00 – 9.33	0.05 – 9.37
% CV inter-assay	7.45 – 13.68	8.66 – 11.06
LOD (ng mL^{-1})	0.76	0.75
LOQ (ng mL^{-1})	2.77	2.85

Supplementary Table S2. HER2 ECD levels in clinical samples measured with *in-house* developed ELISA in comparison to Siemens ADVIA Centaur HER2/neu (upper part) and R&D Systems Quantikine Human ErbB2/HER2 kit (lower part).

HER2 ECD level (ng mL^{-1})		
**ID Patient**	**Reference comparative method: ADVIA Centaur HER2/neu (Siemens)**	***In-house* ELISA as test method**
1	22.60	14.88
2	14.50	15.76
3	18.30	16.47
4	10.80	14.79
5	14.40	14.54
6	11.40	16.01
7	6.30	6.90
8	6.00	7.55
9	5.80	5.58
10	11.20	10.11
11	7.70	6.21
**ID Patient**	**Reference comparative method: Quantikine Human ErbB2/HER2 (R&D Systems)**	***In-house* ELISA as test method**
12	5.37	5.48
13	7.55	8.53
14	25.92	29.68
15	7.60	8.36
16	9.30	9.71
17	6.63	6.10
18	6.43	6.68
19	11.78	11.25
20	9.68	11.98
21	14.20	14.88
22	26.70	20.91

Supplementary Figure S1. Curve demonstrating a linear concentration-response for clinical samples when measuring increasing HER2 ECD levels in serum samples pooling Serum Low HER2 ECD level with Serum High HER2 ECD level in different proportions. Error bars indicate SD of the measurements.

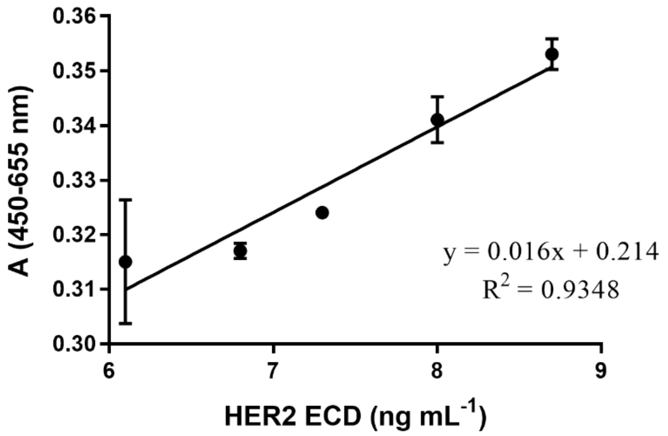

Supplement: Supplementary file 1 — Supplementary Materials. [file 41598_2020_59630_MOESM1_ESM.pdf]
